# Airflow controlled droplet bioprinting enables high-throughput generation of iPSC-derived thymic organoids with *in vivo* T cell functionality

**DOI:** 10.1088/1758-5090/aeae75

**Published:** 2026-10-13

**Authors:** Miranda Poklar, Erin Parlow, Ravikumar K, Nathan Ligenfelter, Mirabella Stump, Elyse Barnes, Lydia Kutzer, Wen Liu, Jiahui Dai, Lauren McCarthy, Carl Engman, Prashant N Kumta, Kimberly F Williams, Yong Fan, Bin Yang, Ipsita Banerjee

**Affiliations:** 1 Department of Chemical and Petroleum Engineering, University of Pittsburgh, Pittsburgh, PA, United States of America; 2 Department of Bioengineering, University of Pittsburgh, Pittsburgh, PA, United States of America; 3 Department of Biomedical Engineering, Duquesne University, Pittsburgh, PA, United States of America; 4 Cancer Institute, Allegheny Health Network, Pittsburgh, PA, United States of America; 5 Department of Biomedical Engineering, Carnegie Mellon University, Pittsburgh, PA, United States of America; 6 Department of Chemical and Biomolecular Engineering, University of Notre Dame, Notre Dame, IN, United States of America; 7 Department of Mechanical Engineering and Materials Science, University of Pittsburgh, Pittsburgh, PA, United States of America; 8 Department of Oral and Craniofacial Sciences, University of Pittsburgh, Pittsburgh, PA, United States of America; 9 McGowan Institute of Regenerative Medicine, University of Pittsburgh, Pittsburgh, PA, United States of America

**Keywords:** induced pluripotent stem cells, droplet printing, thymic epithelial organoids, organ regeneration

## Abstract

The thymus is a critical organ for T cell generation. It is composed of distinct layers of thymic epithelial cells (TECs) that coordinate a complex process of antigen recognition and self-tolerance checks that result in mature populations of T cells. However, it undergoes progressive and permanent involution and loss of functionality which peaks in adolescence, rendering the tissue largely non-functional. Induced pluripotent stem cells (iPSCs) have emerged as promising candidates to generate organoid models of the thymus because of their autologous potential and indefinite self-renewal. Previously our group has shown the capability of generating thymic epithelial progenitor cells (TEPCs) that are able to successfully program T cells *in vitro* and *in vivo* through manual encapsulation of iPSCs in sodium-alginate capsules during differentiation. To translate this capability toward clinically relevant throughput, we present a novel method of air-jetting droplet bioprinting which is capable of producing 21 500 aggregates per milliliter of bioink. We conducted a systematic analysis of critical process parameters governing iPSC aggregation, retention, and differentiation and illustrated the interplay between airflow velocity, which governs capsule size, and crosslinker chemistry governing capsule network stability. We also determined parameters that minimize printing variability and prevent decapsulation of iPSC aggregates during differentiation. Finally, we modeled the growth rate and carrying capacity of different printing configurations, and validated manufactured organoids *in vivo* by generating functional murine T cell populations in athymic nude mice after decapsulation and construction with a decellularized thymus scaffold.

## 1. Introduction

The thymus is the primary site of T cell development and education, acting as the training ground for the adaptive immune system. Yet, paradoxically, it undergoes progressive involution beginning in early life, resulting in the replacement of functional tissue with adipose fat [1]. This degeneration contributes significantly to age-related immune decline (immunosenescence) and increased susceptibility to infections, autoimmunity, and cancer [2]. Despite the urgent clinical need to restore immune function, strategies to regenerate thymic tissue remain limited due to the organ’s biological complexity. The thymus possesses an intricate 3D architecture composed of distinct cortical and medullary regions, populated by specialized thymic epithelial cells (TECs). Cortical TECs (cTECs) are responsible for the positive selection of functional T cells, while medullary TECs (mTECs) drive negative selection to eliminate autoreactive T cells. Recreating this specialized 3D microenvironment is essential for therapeutic success, as conventional 2D monolayer cultures fail to recapitulate the precise spatial organization and cell–cell interactions required for functional T cell selection [3–6].

In the field of personalized cellular therapy, induced pluripotent stem cells (iPSCs) have emerged as an ideal source for generating patient-specific thymic organoids. Because iPSCs are infinitely self-renewing and can be derived autologously, they offer a pathway to bypass immune rejection issues associated with allogeneic transplants [7]. Previous work from our group has established protocols to encapsulate iPSCs in hydrogel [8–14], and further differentiate iPSCs into thymic epithelial progenitor cells (TEPCs). We have demonstrated that these organoids can mature and support T cell development *in vivo* after decapsulation and construction with a decellularized thymus scaffold [15]. However, while functional, these manually generated organoids face a critical translational bottleneck of scalability. It is estimated that recreating a functional native organ requires an average of 1–3 billion cells per milliliter of tissue [16]. Manual encapsulation processes are labor-intensive and prone to batch-to-batch variability, making them incapable of meeting this manufacturing demand. Furthermore, while stirred-tank bioreactors offer scalability, iPSCs are highly sensitive to the high shear rates and hydrodynamic forces inherent in those systems, often resulting in heterogeneous cultures and inefficient differentiation [17–20].

To bridge the gap between laboratory protocols and clinical manufacturing, attention has shifted towards bioprinting as a promising pathway to automate organoid production. However, not all bioprinting modalities are suitable for this application. Standard extrusion bioprinting, which prints ‘cell pastes,’ often suffers from low throughput; for example, previous studies utilizing extrusion printing for kidney organoids could only achieve production rates of roughly 18 organoids per minute [21]. Furthermore, recent experience from our group with extrusion bioprinting of islet organoids has demonstrated similar results in terms of throughput [22]. In contrast, droplet bioprinting, specifically coaxial air-jet systems, offers a promising alternative for mass production. By utilizing a pressurized air stream to disrupt surface tension and drive the formation of microscale droplets, this method enables the rapid generation of uniform hydrogel capsules without exposing cells to damaging shear stress [23]. Additionally, these microscale capsules possess high surface-to-volume ratios, facilitating the efficient transport of oxygen and nutrients necessary to support high-density cell aggregates during prolonged differentiation cultures.

In this study, we present the development of a scalable, airflow-controlled droplet bioprinting system optimized to produce iPSC-derived thymic epithelial organoids. We moved beyond simple encapsulation to perform a systematic analysis of the critical bioprocessing parameters governing aggregate formation, retention, and differentiation. Specifically, we investigated the interplay between airflow velocity, which dictates production throughput, and crosslinker chemistry, which governs hydrogel network stability. We identified a critical trade-off between production speed and batch consistency, discovering that high-throughput printing (5 l min^−1^) introduced more variability compared to optimized lower-flow conditions (2 l min^−1^). Furthermore, we demonstrate that optimizing the hydrogel crosslinking network is critical to preventing the ‘decapsulation’ phenomenon, where growing aggregates escape the hydrogel matrix making them unavailable for downstream differentiation. We also investigate crosslinker dependent effects on the final differentiation phenotype of iPSC-TEPCs (cTEC vs mTEC) through patterns of EpCAM and GP38 expression. Finally, we validate the system by integrating logistic growth modeling of aggregate expansion and demonstrate that these bioprinted organoids generate functional TEPCs capable of supporting T cell development *in vivo*.

## 2. Materials and methods

### 2.1. Bioink preparation

For all bioinks prepared, low viscosity alginate (*G*/*M* ratio ∼1.56, MilliporeSigma, W201502) at 1.6–2.0% w/v was mixed with DMEM/F12 (Gibco) to form a hydrogel bioink. To form the ink, the appropriate amount of alginate was added to DMEM/F12 and mixed for one hour. After mixing, the bioink was sterilized through 0.22 *μ*m syringe filters (MilliporeSigma) and stored at 4 °C until needed.

### 2.2. General printing of alginate bioink

Alginate bioink was placed at room temperature (RT) for 15 min prior to printing. The bioink was then transferred to a 10 ml syringe, and a ¼’ 30 G needle (Allevi) was used for printing. Once the needle was added, a custom-designed resin-printed air jacket was fitted around it, allowing the outlet of the needle to sit 1 mm below the outlet of the air jacket. This air jacket was supplied by an external tank of medical grade compressed air (AI USPEA, Airgas), where the flow rate was varied between 1 and 6 l min^−1^. After ensuring a proper fit of the air jacket, the syringe was placed vertically in a syringe pump (NE-1000, SyringeONE), so that the outlet of the needle faced the ground. Below the needle outlet resided a 250 ml beaker containing CaCl_2_, SrCl_2_, or BaCl_2_ at the specified concentration in deionized water at a volume of 100 ml on a stir plate (Cole-Parmer) used for capsule collection and crosslinking. Crosslinking is achieved when divalent cations from the crosslinkers interact with two distinct strands of the alginate bioink polymer creating a mesh network. Alginate interaction with divalent cations displaces monovalent sodium ions. In general, it is known that as the atomic radius of the divalent cations increases, stronger, stiffer networks of hydrogel are formed.

The distance from the outlet of the needle to the beaker volume surface was maintained at 13.5 cm. An extrusion rate of 0.09 ml min^−1^ (crosslinking in CaCl_2_) or 0.16 ml min^−1^ (SrCl_2_, BaCl_2_) was used (table 1). The stir plate was set at either 350 RPM (CaCl_2_), or 250 RPM (SrCl_2_, BaCl_2_). Matching ink extrusion rates and stir speeds enabled maintenance of sphericity between differing crosslinkers. Capsules were printed for 4–5 min and crosslinked for 1.5 min. Following, the capsules were removed and washed three times in PBS (Gibco). Capsules were imaged with an inverted microscope (IN480, Amscope) and diameters were quantified using ImageJ.

**Table 1.** Ink extrusion rates and stir speeds for different crosslinkers.

Crosslinker	Ink extrusion rate (ml min^−1^)	Stir speed (RPM)
100 mM CaCl_2_	0.09	350
200 mM CaCl_2_	0.09	350
70 mM SrCl_2_	0.16	250
35 mM BaCl_2_	0.16	250

### 2.3. Real-time monitoring of droplet generation

A camera-based monitoring system was used to quantify droplet generation rate in real time. For each alginate concentration and crosslinking condition, the generation rate was quantified from 3 separate 1 second video clips, following the printing protocol described in section 2.2. The mean and standard deviation of the generation rate was calculated from these 3 video clips. The imaging system consisted of a monochrome camera (acA1920-155u, Basler) and a C-mount lens with a focal length of 25 mm. An extension tube measuring 1 cm was inserted between the camera and the lens to reduce the minimum focusing distance to approximately 10 cm, facilitating image acquisition. The camera captured droplet generation at 327 frames per second, which was achieved by restricting the sensor region to 1920 × 150 pixels. The continuous droplet generation and separation produced local intensity variations. A custom MATLAB script was developed and used to perform real-time rate quantification through analysis and tracking of the intensity variations. The MATLAB script is available via GitHub in the following link:

https://github.com/parlowe/Biofabrication_ Submission_BF106576.

### 2.4. iPSC 2D culture and cell-bioink preparation

All iPSCs used in the study were the progeny of one iPSC line, Y1 generated in house from male dermal fibroblast cells. The pluripotency and differentiation efficiency of this cell line has been demonstrated by our group in previous studies [15, 22]. 2D undifferentiated iPSCs were cultured to confluency with mTeSR^+^ (STEMCELL Technologies), and dissociated to single cell form using a modified protocol previously established by our group [11]. Undifferentiated 2D iPSCs were first incubated overnight in mTeSR^+^ with 10 *μ*M Y-27632 (ROCK Inhibitor; RI) (R&D Systems). The next day cells were incubated with Accutase (Life Technologies) for 7–10 min and manually pipetted to obtain a single cell suspension. Cells were then filtered through a 40 *μ*m cell strainer (Falcon) to remove any remaining colonies and counted. Cells were suspended in the alginate bioink by addition of 1 ml alginate per 1 million cells and incubated for 45 min to ensure coating of all cells in the bioink.

### 2.5. iPSC-capsules and culture

For cells intended for studies monitoring undifferentiated iPSC expansion and pluripotency, alginate capsules (1.6–2.0% w/v) were crosslinked in either 100 or 200 mM CaCl_2_ and printed with an airflow rate 4–6 l min^−1^. After printing and crosslinking, capsules were cultured in mTeSR^+^ with 10 *μ*M RI for four days, with media changes occurring every other day. Phase images were taken with an Olympus CKX41 each day over the culture period to track aggregate size and number, quantified using ImageJ.

For studies involving thymic epithelial progenitor cell (TEPC) differentiation within the capsules, 2.0% w/v alginate capsules were crosslinked in either 100 mM CaCl_2_, 200 mM CaCl_2_, 70 mM SrCl_2_, or 35 mM BaCl_2_. All capsules were printed with either an airflow rate of 2 l min^−1^ or 5 l min^−1^. For cell-embedded capsules printed at 2 l min^−1^, the capsules were cultured for 3 d in mTeSR^+^ with 10 *μ*M RI before switching to the appropriate media for differentiation. For cell-embedded capsules printed at 5 l min^−1^, the capsules were cultured for 2 d in mTeSR^+^ with 10 *μ*M RI before switching to the appropriate media for differentiation. The TEPC differentiation protocol is detailed in section 2.6. Phase images were taken with an Olympus CKX41 each day over the incubation period to track aggregate size.

A general estimate of the average number of aggregates and cells produced in each printing study was also calculated, using phase imaging. To determine the average number of aggregates produced per ml of bioink, the average number of capsules produced per ml of bioink was first calculated (equation (1)),

\begin{align*} &amp;\frac{\mathrm{Total}\ \#\ \text{of beads generated}}{\text{ml bioink}}\nonumber\\ &amp;\quad = \frac{\mathrm{1}}{\text{Average individual bead volume}\left(\mathrm{ml}\right)}.\end{align*}

Using the phase imaging at the end of differentiation, the average number of aggregates per capsule was calculated. This was then used to find the total number of aggregates produced, which could be normalized per ml of bioink (equation (2)),

\begin{align*} &amp; \#\ \text{Aggregates generated/ ml bioink}\nonumber\\ &amp;\quad = \frac{\#\ \mathrm{Aggregates}}{\mathrm{Bead}}*\frac{\mathrm{Total}\ \#\ \text{beads generated}}{\text{ml bioink}}.\end{align*}

The coefficient of variance (CV) was calculated by dividing the standard deviation of the average number of aggregates generated per ml of bioink by the mean of the average number of aggregates generated per ml of bioink.

### 2.6. iPSC differentiation into thymic epithelial progenitor cells

Using a modified version of a previously published protocol TEPCs were generated using the stagewise differentiation steps as described below [3]. Stage 1 for definitive endoderm was carried out following a beta cell differentiation protocol [24]. The stage 1 base media was composed of 2.44 mM D-Glucose (Gibco), 2.46 g l^−1^ NaHCO_3_ (MilliporeSigma), 2% FAF-BSA (Fisher Scientific), 2 mM Glutamax (Gibco), 1% Pen/Strep (Lonza), 0.25 mM Vitamin C (MilliporeSigma), 1:50 ITS-X (MilliporeSigma), and 490 ml MCDB131 (Corning/Gibco). Stage 2 base media for anterior foregut endoderm was Roswell Park Memorial Institute (RPMI) media supplemented with 0.5% B27 (Gibco). Stages 3 and 4 base media for ventral pharyngeal endoderm and thymic epithelial progenitor was DMEM/F12 (Gibco) media supplemented with 0.5% B27.

Media changes were completed as follows with supplements to the appropriate base media. Stage 1 (Days 1–3): 100 ng ml^−1^ Activin A (R&D Systems) and 3 *μ*M Chir99021 (Stemgent) (Day 1 only). Stage 2 (Day 4): 100 ng ml^−1^ Activin A, 0.25 *μ*M retinoic acid (Millipore Sigma). Stage 2 (Days 5–6): 0.25 *μ*M retinoic acid, 50 ng ml^−1^ BMP4 (Miltenyi Biotec), and 5 *μ*M LY364947 (MilliporeSigma). Stage 3 (Days 7–8): 0.1 *μ*M retinoic acid, 5 *μ*M LY364947 (Days 7–8 only), 50 ng ml^−1^ BMP4, 50 ng ml^−1^ Wnt3A (R&D Systems), 50 ng ml^−1^ FGF8b (Miltenyi Biotec), and 0.5 *μ*M KAAD-cyclopamine (MilliporeSigma). Stage 4 (Days 9–10) supplements matched Stage 3 with the exclusion of LY364947. (Days 11+): DMEM/F12 supplemented with 0.5% B27, no additional growth factors.

### 2.7. Live/dead staining of printed cell beads

Cell viability was visualized using a viability kit (Invitrogen). The live cell stain Calcein AM was added at 1:4000, and the dead cell stain Ethidium Homodimer-1 (Ethd-1) was added at 1:1000. Both stains were added to the intact capsules and incubated at room temperature for 1 h. Excess stain was then removed, and samples were imaged on a Zeiss Axio Imager 2. Aggregates were then harvested from the capsules by applying EDTA (100 mM) for 3 min and gently stirred to break apart any remnants of the bioink. The aggregates were washed with PBS and then imaged in the same manner as the intact cell-embedded capsules. Aggregate size, dead cell distribution, and percent viability were quantified in ImageJ.

The percent viability of an aggregate was calculated from the ratio of area labeled green to the sum of the live (green) and dead cell area (red). To estimate the actual dead cell area, a correction factor of 2.3 (ratio of cell area to nuclei area) was applied to the area labeled red. The dead cell distribution in the aggregates was visualized by plotting the distance between red labeled regions (dead cell nuclei) and the centroid of the aggregate (*r*) with respect to the radius of the aggregate (*R*). *r*/*R* closer to zero indicates the presence of dead cells closer to the core of the aggregate while the ratio being closer to one indicates that the dead cells are closer to the periphery of the aggregate.

### 2.8. Immunofluorescence staining of printed cell capsules

Following bioprinting and culture, cells were harvested by dissolving the alginate with EDTA. The aggregates were fixed overnight in 4% w/v paraformaldehyde at 4 °C. Aggregates were then washed with PBS and primary antibodies were added after dilution in staining buffer (10% Donkey serum and 1% Triton X-100 in PBS) at 1:200 and incubated overnight at 4 °C. Secondary antibodies (1:500) were then added after washing with PBS, and also incubated overnight at 4 °C. The next day, aggregates were mounted on a glass slide using Gold Antifade with DAPI and then imaged on an Olympus Fluoview confocal microscope. Primary antibodies for OCT4 (Mouse, R&D Systems) and NANOG (Goat, R&D Systems) along with secondary antibodies anti-mouse Alexa Fluor 647 and anti-goat Alexa Fluor 488 were used for imaging undifferentiated iPSC aggregates. Primary antibodies for EpCAM (Rabbit, R&D Systems) and KRT8 (Mouse, R&D Systems) along with secondary antibodies anti-mouse Alexa Fluor 488 and anti-rabbit Alexa Fluor 555 were used for imaging TEPC aggregates.

### 2.9. Flow cytometry of printed cell capsules

Following bioprinting and differentiation to the TEPC stage (detailed in 2.5 and 2.6), cells were examined for surface marker expression. Aggregates were harvested from the alginate capsules using EDTA. Aggregates were further dissociated into single cells using TrypLE (Gibco) for 8–10 min and strained using a 40 *μ*m nylon filter to obtain a single cell suspension of TEPCs. A subsection of cells was set aside to act as an unstained control, and all other cells were incubated with near-IR dye (1 *μ*l ml^−1^) at RT for 10 min (Invitrogen). Cells were washed with flow staining buffer (FSB—Invitrogen) and then incubated for 10 min with human Fc block (0.5 mg ml^−1^—Invitrogen). A subsection of cells were then set aside to act as a near-IR control, with all other cells stained at RT for 15 min with the following antibodies: Pacific Blue™ anti-human CD326 (EpCAM—Biolegend), APC anti-human CD205 (Biolegend), GP38-AF488 (BD Biosciences), and PE anti-human Delta-like protein 4 (DLL4—Invitrogen). All cells were then washed two times with FSB and fixed for 10 min with IC fixation buffer (Invitrogen). Following this, samples were washed and resuspended in FSB for flow cytometry. Samples were run in a BD FACS Aria flow cytometer. All analyses were conducted with FlowJo v10.10.

### 2.10. Logistic growth modeling

To investigate the entire printing parameter space, and identify optimal conditions for reproducible TEPC production, we applied logistic growth modeling to the aggregate growth dynamic data collected over time for all material and airflow conditions. The logistic growth model describes aggregate expansion through three parameters (equation (3)).

\begin{equation*}A\left( t \right) = \frac{K}{{\left( {1 + \frac{{K - {A_0}}}{{{A_0}}}{{\mathrm{e}}^{ - rt}}} \right)}}{ }\end{equation*}

where *A*_0_ describes the initial aggregate size in *μ*m, *K* represents the carrying capacity of a capsule in *μ*m, and *r* is the rate parameter in units of d^−1^. Curve fitting was completed using SciPy (version 1.16.3) in Python (version 3.13.9). Convergence criteria were the defaults specified by SciPy for curve fitting.

### 2.11. Ethical statement

All the works in the study were reviewed and approved by the Institutional Animal Care and Use Committee (IACUC) at Allegheny Health Network Research Institute.

### 2.12. Mouse implantation with printed TEPCs

Inbred athymic nude mice were purchased from the Jackson Laboratory (Nu/J, Strain # 002019). Mice were housed in pathogen-free optimal environmental conditions, including a 14 hour light/10 hour dark cycle, 18 °C–24 °C temperature and 40%–60% humidity.

Single iPSCs were dissociated, printed, and cultured as described in 2.4, 2.5, and 2.6. Cells were printed in 2.0% w/v alginate at a flow rate of 2 l min^−1^ and crosslinked in 200 × 10^−3^ M CaCl_2_. After completion of differentiation aggregates were decapsulated from the alginate beads using EDTA. TEPC aggregates were seeded into decellularized murine thymus scaffolds along with *in vitro* generated human CD5 + CD7+ progenitor T cells (Pro-Ts) at a 5:1 ratio to generate the thymus organoids, as described previously [15, 25]. Thymus organoids were cultured for 7 d before transplantation in 24 well transwells. Recipient mice were anesthetized and the iPSC-TEPC + Pro-T thymus organoids were surgically implanted beneath the kidney capsule.

After 45 d, mice were sacrificed, and the graft, spleen, and mesenteric lymph nodes were harvested and digested. Single cells were stained for murine lymphoid and T cell markers including CD45 (BD Horizon), CD3 (BD Pharmingen), CD4 (BD Horizon), and CD8 (BD Pharmingen). Digested tissue samples underwent flow cytometry and were first gated to isolate murine CD45^+^ CD3^+^ positive populations. From this population, sub-populations of murine CD4 and CD8 positive cells were identified.

### 2.13. Statistical analysis

All quantitative data was expressed as mean ± standard deviation, unless stated otherwise, and plotted using GraphPad Prism 10. Data were tested for statistical differences using unpaired *t*-tests except for tests of significance in logistic growth modeling parameters which used Wald Tests completed using the SciPy package for Python 3. Statistical significance was considered for *: *p* < 0.05, **: *p* < 0.01, ***: *p* < 0.005, and ****: *p* < 0.001.

Measurements of aggregates or capsules were obtained from multiple technical observations within a given printing experiment. The precise number of measurements is provided in the relevant figure captions. For flow cytometry characterization and analysis, reported proportions are derived from a single representative flow experiment. Finally, *in vivo* results are presented for three experimentally treated mice and two control mice.

## 3. Results

### 3.1. An airflow controlled bioprinting system facilitates microscale droplet formation

#### 3.1.1. Development of coaxial airflow bioprinting system

Our initial focus was on developing and optimizing a system capable of mass-producing homogenous, high-volume alginate capsules to encapsulate iPSCs for organoid production. These capsules require spherical geometry, uniform dimensions, and culture stability to support differentiation and organoid generation. We developed an extrusion-based droplet bioprinting system wherein the alginate bioink was extruded via vertically oriented syringe pump through a needle into a stirring crosslinking bath, where the droplets undergo polymerization and stabilize as described in section 2.2 (figure 1(A)). The droplet size was regulated via air-jetting using a custom-designed, resin-printed air jacket positioned at the base of the needle, with medical grade airflow controlled by a flowmeter (figure 1(B)). Further details of this setup can be found in supplemental figures S1 and S2. The applied airflow disrupts the surface tension of the extruding alginate, generating microdroplets at the needle tip, which were subsequently released into the crosslinking bath. This process was captured by high-speed imaging, as depicted in figure 1(C) (one frame extracted from supplementary video 1). Following this initial system establishment, we focused on achieving controlled droplet formation and scalability.

**Figure 1.**
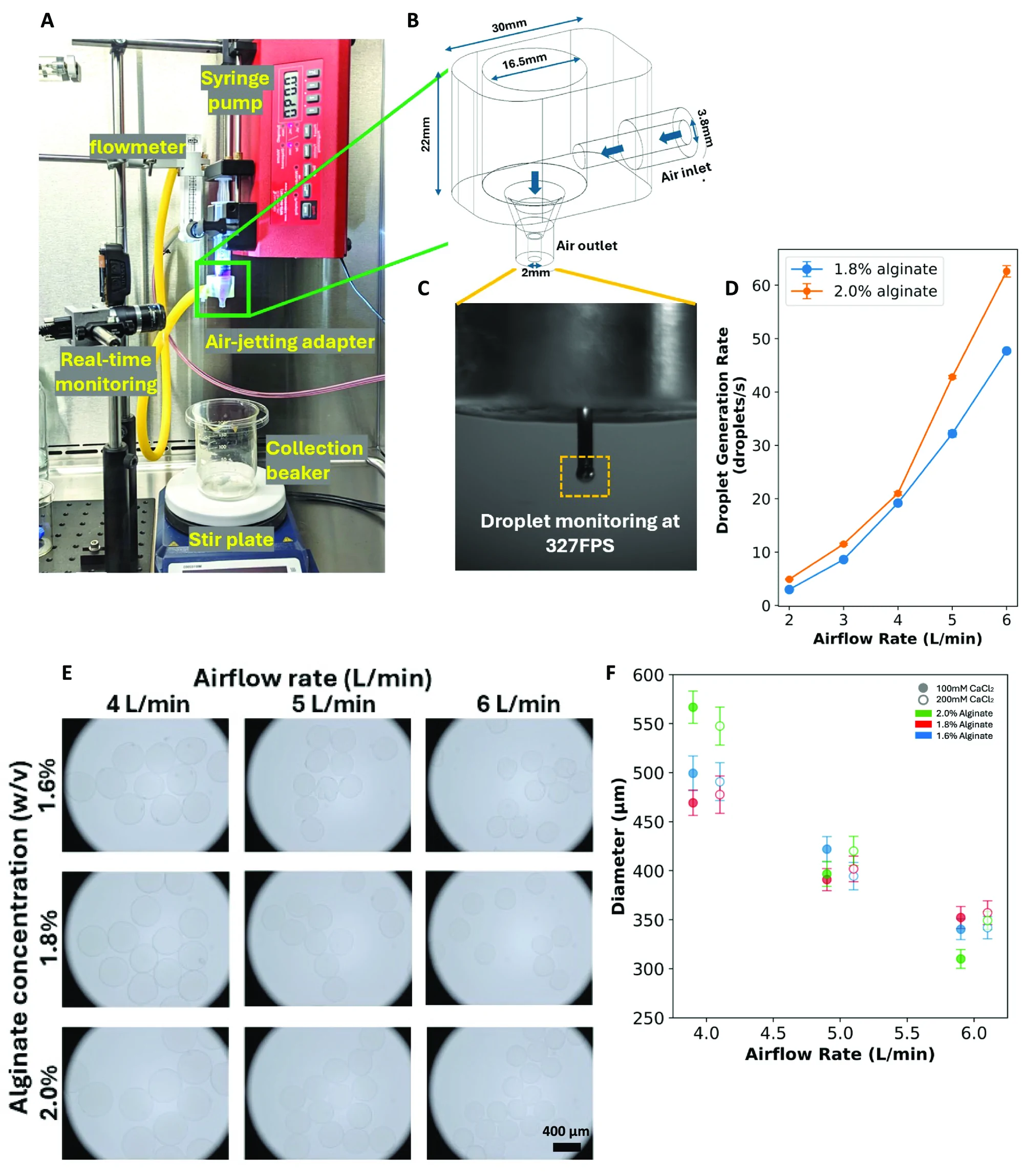
(A) Picture of assembled system with labeled components (B) 3D model of the air-jetting adapter highlighting the air inlet and outlet. (C) One frame extracted from supplementary video 1 captured by a high-speed camera demonstrating a droplet being formed. (D) Capsule generation rate (capsules s^−1^) for 1.8% (blue) and 2.0% w/v (orange) alginate printed at airflow speeds 2–6 l min^−1^. (E) Representative images of capsules crosslinked in 100 mM CaCl_2_, in various alginate concentrations and various airflow rates. Scale bar represents 400 *μ*m (F) Average capsule diameter of alginate at the indicated concentration (1.6% blue, 1.8% orange, and 2.0% green) when printed at 4–6 l min^−1^ and crosslinked in 100 mM CaCl_2_ (solid) or 200 mM CaCl_2_ (open). (n = minimum 78 capsules).

#### 3.1.2. Airflow mediated control of droplet dimensions and generation kinetics

In order to assess the impact of airflow on capsule generation, real-time video analysis was used to quantify the generation rates of the capsules across various airflow velocities for two alginate concentrations. The capsule generation rate increased significantly with the airflow rate, for both the alginate concentrations tested (figure 1(D)). At 1.8% w/v alginate, generation rate increased from 3.0 ± 0.1 droplets s^−1^ at 2 l min^−1^ to 48 ± 0.4 droplets s^−1^ at 6 l min^−1^. Similarly, 2.0% w/v alginate demonstrated increased generation from 5 ± 0.1 droplets s^−1^ at 2 l min^−1^ to 63 ± 1.1 droplets s^−1^ at 6 l min^−1^. Significant differences between the alginate concentrations emerged only at higher airflow velocities of 5 and 6 l min^−1^, where 2% w/v alginate produced significantly more capsules than 1.8% w/v alginate. This observation is likely due to transition to turbulent airflow states at 5 and 6 l min^−1^ (Reynolds numbers 3500 and 4200 respectively) which causes a higher number of droplets due to shear forces. Importantly, minimal standard deviations across all conditions indicate highly reproducible and consistent generation rates.

While bead generation rate is critical for achieving high organoid throughput, capsule dimensions are equally important for the organoid formation process. Quantitative analysis of capsule diameter confirmed that increasing the airflow rate significantly reduced capsule diameter across all printing conditions (figure 1(E)). Representative images of alginate capsules (1.6–2.0% w/v), crosslinked in 100 mM CaCl_2_ (figure 1(F)), and printed at 4–6 l min^−1^ illustrate this size reduction and improved spherical uniformity at higher airflow rates. Notably, neither alginate nor CaCl_2_ concentrations substantially influenced the overall capsule diameter, as evidenced by clustering of average capsule diameters at each of the airflow rates tested, further confirming airflow to be the primary control parameter of the system.

#### 3.1.3. Rheological effects of cellular inclusion on droplet formation

We next investigated whether the air-jetting system could maintain critical biological functions with bio-printed iPSCs. Bioprinting of iPSCs presents a fundamental challenge: for scalability it is preferable to print the iPSCs as single cells, however iPSCs are unstable as single cells and require cell–cell contact for survival and effective differentiation. Thus, our approach was to bio-print the iPSCs as single cells within the alginate ink, hypothesizing that appropriate biophysical cues within the alginate microsphere will induce aggregation and subsequent differentiation. Survival of bio-printed single cell iPSCs was ensured by supplementing with RI until cellular aggregation was visible.

Undifferentiated iPSCs were dissociated into single cells, resuspended in alginate bioink at varying concentrations and subjected to air-jetting using parameters optimized for cell-free bioink (figure 2(A)). Since cellular inclusion altered the rheological properties of the bioink, printing parameters were re-evaluated first using 100 mM CaCl_2_ as the crosslinking agent [26]. The presence of cells significantly influenced droplet formation dynamics in a concentration-dependent manner. At 1.6% w/v alginate, all the tested airflow rates (4–6 l min^−1^) resulted in irregular, non-spherical droplets (figures S3(A)–(C)), a phenomenon not observed in the cell-free blank controls. While aggregate formation could be visualized by Day 2 of culture, the morphological inconsistency excluded 1.6% w/v alginate as a viable candidate for cellular bioprinting.

**Figure 2.**
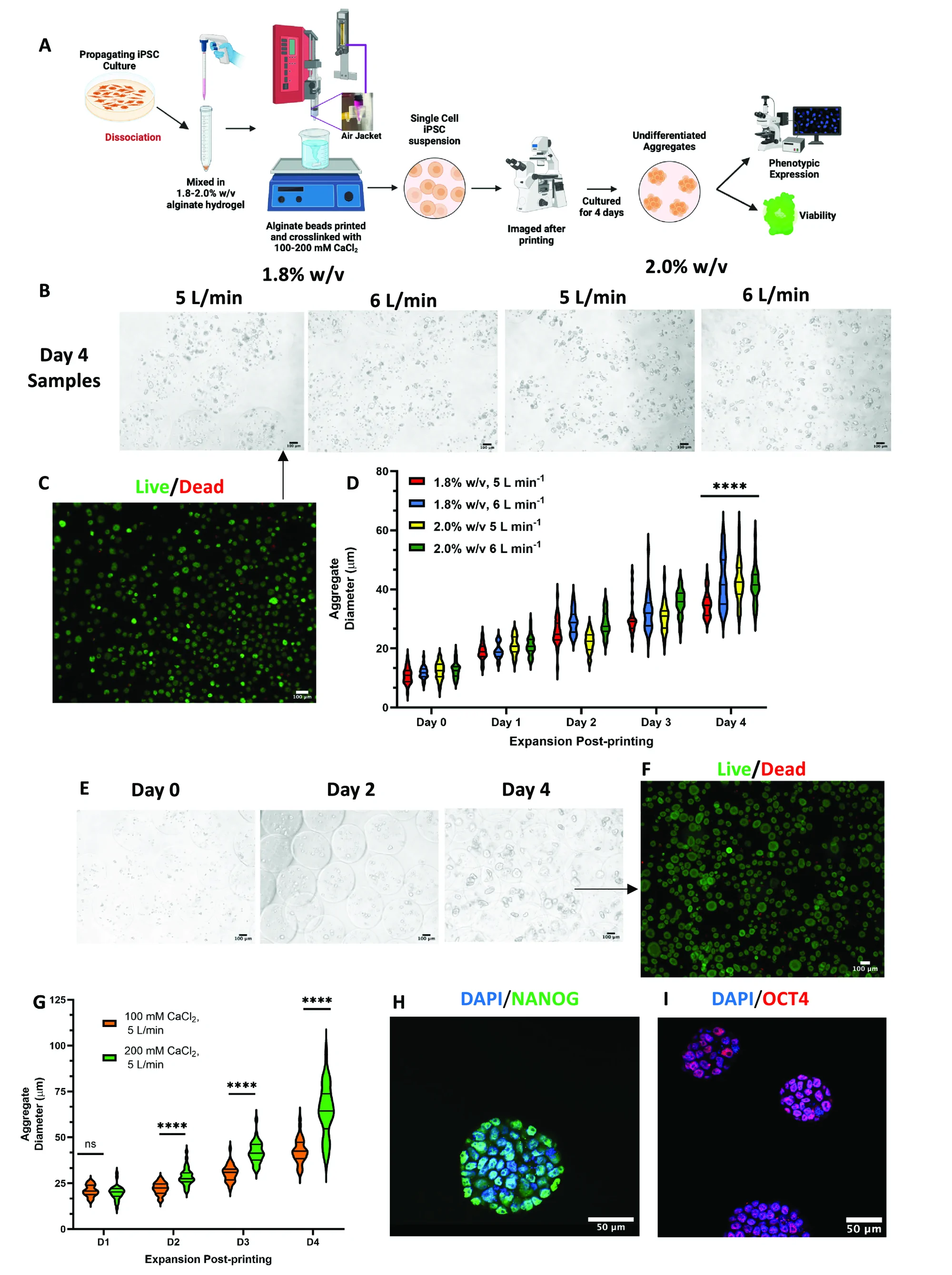
(A) Schematic view of printing iPSCs embedded as single cells in a 1.8–2.0% w/v alginate, seeded at 1 million cells: 1 ml alginate, airflow speed 4–6 l min^−1^, and crosslinking concentration of 100–200 mM CaCl_2_. After printing, cells were cultured for four days, observed for aggregation, and then tested for pluripotency and viability. Created in BioRender. Parlow, E. (2026) https://BioRender.com/ncff8cw (B) Phase images of printed iPSC-embedded capsules crosslinked in 100 mM CaCl_2_, 4 d after printing. (C) Live/Dead staining of aggregates printed in 1.8% w/v alginate, and an airflow speed of 5 l min^−1^. Staining is performed after harvesting the aggregates from alginate. Live cells are shown in green, dead cells are shown in red. (D) Aggregate size distribution measured for 4 d of expansion for conditions shown in (B). Aggregates were imaged each day using phase microscopy (*n*= minimum 30 aggregates, ****: *p* < 0.001). Key significance values noted, though other comparisons may also be significant. For figures (E)–(I), all aggregates were cultured in 2.0% w/v alginate, airflow of 5 l min^−1^, and crosslinked in 200 mM CaCl_2_. (E) Phase images of printed iPSC-embedded capsules at 0, 2, and 4 d of expansion. (F) Live/Dead staining of aggregates on Day 4, staining is performed after harvesting the aggregates from alginate. Live cells are shown in green, dead cells are shown in red. (G) Comparative aggregate size (diameter) distribution for capsules crosslinked in 100 mM or 200 mM CaCl_2_, measured for 4 d of aggregate expansion. Cells were imaged each day using phase microscopy (*n* = minimum 30 aggregates, ns: not significant, **** *p* < 0.001). (H) Printed undifferentiated aggregates were fluorescently stained for nuclei (DAPI), and pluripotency marker NANOG (green). Scale bar representing 50 *μ*m. (I) Printed undifferentiated aggregates were fluorescently stained for nuclei (DAPI), and pluripotency marker OCT4 (shown in red). Scale bar representing 50 *μ*m. All other scale bars are 100 *μ*m.

In contrast, higher alginate concentrations of 1.8% (figures S4(A)–(C)) and 2.0% (figures S5(A)–(C)) w/v alginate maintained spherical morphology across all tested airflow rates, suggesting increased polymer density compensated for cell-induced rheological changes. At high airflow rate of 6 l min^−1^, a small subpopulation of undersized ‘overspray’ microcapsules was observed, while the majority of capsules were of a consistent size. Notably, inclusion of cells in the bioink resulted in increased capsule diameter over cell-free blank capsules, with this effect being inversely proportional to alginate concentration, with larger increases seen at 1.8% alginate compared to 2.0% alginate (figure S6). This is likely due to the decreased viscosity of lower concentration bioinks allowing for inclusion of larger numbers of iPSCs. The inverse relationship between airflow rate and capsule diameter remained consistent with the cell-free trends.

### 3.2. iPSC bioprinting preserves pluripotency while enabling controlled aggregation with alginate microspheres

Droplet morphology, aggregation kinetics and pluripotency maintenance were assessed over a 4 d culture period (figure 2(A)). Temporal analysis of cell aggregation dynamics revealed rapid self-assembly kinetics across all viable printing conditions. Initial cell–cell contact was established within 48 h, followed by progressive expansion through Day 4 (figure 2(B)). Live/Dead staining demonstrated high aggregate viability for 1.8% alginate printed at 5 l min^−1^ (figure 2(C)) Quantitative morphometric analysis demonstrated comparable growth dynamics resulting in comparable aggregate sizes across most conditions by Day 4 (figure 2(D)), with the notable exception of 1.8% w/v alginate printed at 5 l min^−1^, which produced significantly smaller aggregates (*p* < 0.001). Importantly, this reduction in aggregate size did not reflect compromised viability, as Live/Dead staining confirmed minimal cell death both within the encapsulated capsules (figure S7) and following harvesting from capsules (figure 2(C)). Increasing the airflow rate to 6 l min^−1^ did not compromise cell viability (figure S8), indicating that the shear forces at higher airflow rates remain within tolerable limits for iPSCs.

The mechanical properties of alginate hydrogels are primarily governed by crosslinking chemistry, which directly influences substrate stiffness and porosity, which are parameters known to influence iPSC phenotype and viability [27–33]. Hence, we evaluated the effect of crosslinking concentration on the cell aggregate dynamics and pluripotency. Doubling the CaCl_2_ concentration from 100 to 200 mM CaCl_2_ did not significantly alter capsule dimensions (498 ± 21 *μ*m at 200 mM CaCl_2_ vs 489 ± 17 *μ*m at 100 mM CaCl_2_ *p* = 0.423, figure S9) for 2.0% alginate. Both crosslinking conditions supported robust cell aggregation (figures 2(E) and S10) with initial cluster formation occurring within 48 h. Crosslinking at 200 mM CaCl_2_ retained high viability and aggregate morphology upon harvesting after 4 d post printing (figure 2(F)). However, aggregates in the 200 mM condition exhibited accelerated expansion, reaching 65 ± 14 *μ*m by Day 4 (figure 2(G)) compared to smaller aggregates at 100 mM, suggesting that the biophysical cues provided by higher crosslinking density are more conducive to aggregate growth.

Crucially, these variations in mechanical environment and growth kinetics did not compromise the pluripotency. Immunostaining confirmed robust expressions for both OCT4 and NANOG, which were distinctly localized in the nucleus, as demonstrated with co-localization with DAPI (figures 2(H) and (I)). The maintenance of pluripotency despite differential mechanical environments and growth patterns underscores the robustness of our bioprinting platform and its compatibility with downstream differentiation. These findings establish our bioprinting platform as robust for scalable organoid production, with crosslinking density serving as an additional tunable parameter for modulating aggregate growth without sacrificing pluripotency.

### 3.3. Crosslinking chemistry governs aggregate retention during directed differentiation

Having demonstrated robust formation of pluripotent aggregates from bio-printed single iPSCs, we next evaluated directed differentiation capability. We adopted our previously established protocol for generating thymic epithelial progenitor cells (TEPCs) [15], involving a propagation phase followed by staged differentiation (figure 3(A)).

**Figure 3.**
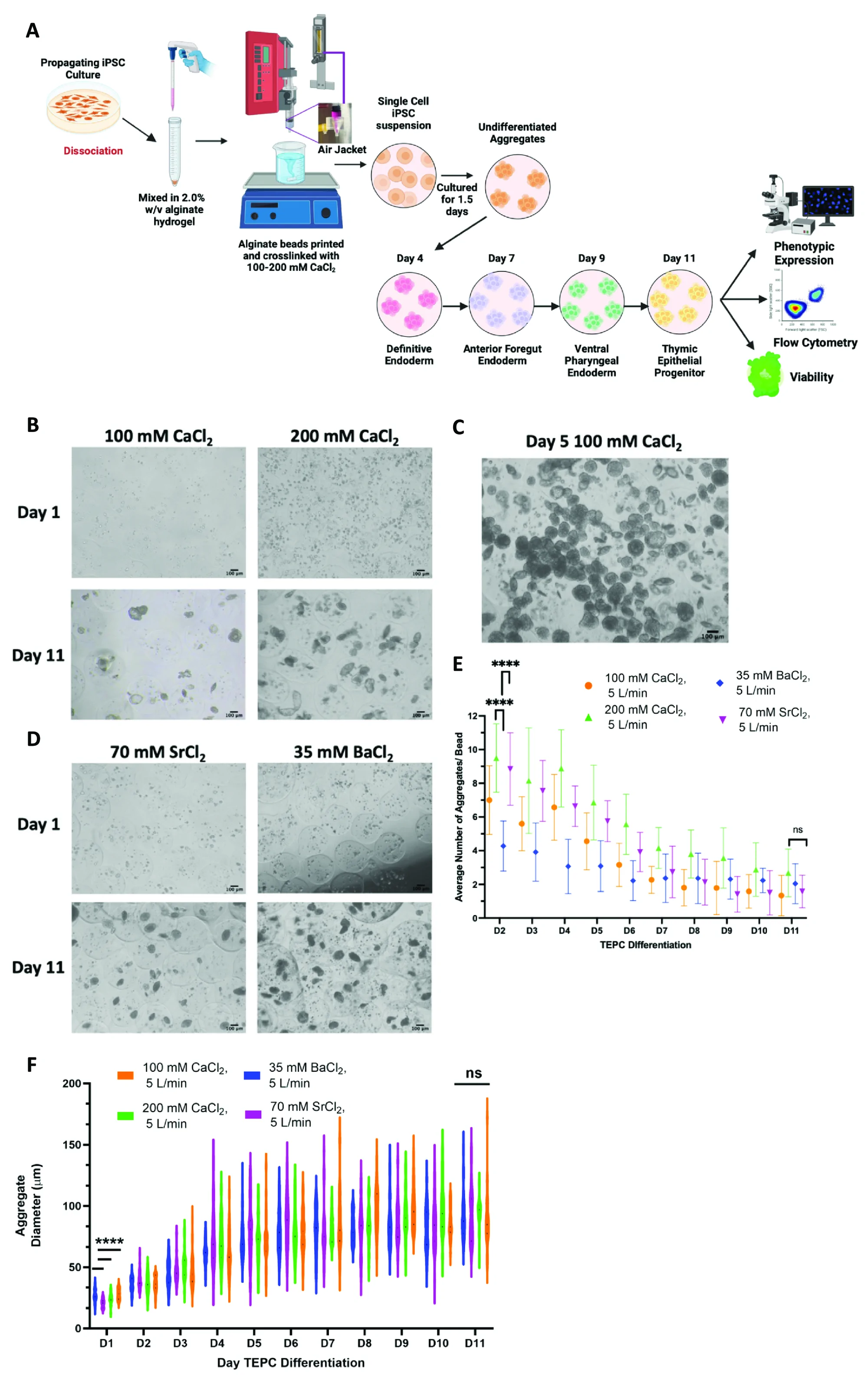
(A) Schematic view of printing iPSCs and differentiating to a thymic epithelial cell progenitor lineage using a syringe air jacket bioprinting system. Created in BioRender. Parlow, E. (2026) https://BioRender.com/phhcxyk (B) Phase images of printed iPSC-embedded capsules crosslinked in 100 mM CaCl_2_ and 200 mM CaCl_2_ at 5 l min^−1^ airflow speed. Images taken 1 and 11 d into thymic epithelial progenitor (TEPC) differentiation. (C) Phase image taken of printed iPSC-embedded capsules crosslinked in 100 mM CaCl_2_, 5 d into thymic epithelial progenitor cell (TEPC) differentiation, demonstrating decapsulation occurring during differentiation. (D) Phase images of printed iPSC-embedded capsules crosslinked in 70 mM SrCl_2_ and 35 mM BaCl_2_ at 5 l min^−1^ airflow speed. Images taken 1 and 11 d into TEPC differentiation. (E) Average number of aggregates/capsule at specific day of differentiation, starting from Day 2 (*n* = minimum 10 capsules). Significance values at the beginning and end are noted. (F) Average aggregate diameter during TEPC differentiation in capsules crosslinked in 100 mM CaCl_2_, 200 mM CaCl_2_, 70 mM SrCl_2_, and 35 mM BaCl_2_ at 5 l min^−1^ airflow speed (*n* = minimum 27 aggregates,****: *p* < 0.001. ns: non-significant). Significance values at the beginning and end are noted, though other comparisons may also be significant. Scale bars of all phase images represent 100 *μ*m.

Initial attempts using the 4 d propagation period resulted in excessive aggregate growth and subsequent ‘decapsulation’ from the hydrogel droplets (data not shown). Notably, the capsules remained intact following decapsulation (data not shown) while the aggregates remaining in the capsules continued growth and differentiation. To mitigate premature decapsulation, the propagation phase was shortened to 2 d before inducing differentiation, effectively limiting aggregate expansion. Additionally, based on results demonstrated previously, we found that encapsulation in 2.0% alginate with a 5 l min^−1^ printing speed proved to be the optimal printing conditions for cell-laden printing. These parameters resulted in sufficient aggregate growth during expansion, while also maintaining manufacturing consistency in geometry of the printed capsules. For all directed differentiation experiments, these printing parameters were used.

#### 3.3.1. Crosslinker identity modulates aggregate retention

Under both 100 mM and 200 mM CaCl_2_ crosslinking conditions, aggregates were visible by Day 1 of differentiation and continued expanding throughout the differentiation period (figure 3(B)). Phase images can be found in figures S11 (100 mM) and S12 (200 mM). However, by approximately Day 5 of differentiation, during the anterior foregut specification stage, extensive decapsulation was observed in the 100 mM CaCl_2_ condition, with large clusters of decapsulated aggregates dispersed throughout the media. (figure 3(C)). This phenomenon was less frequent in the 200 mM CaCl_2_ condition, indicating increased crosslinking enhanced aggregate retention. Only aggregates that remained within the capsule were considered for subsequent analysis.

Given this concentration dependent effect, we hypothesized that crosslinker identity, specifically cation binding affinity to alginate, might provide additional control over retention. Capsules were crosslinked using 35 mM BaCl_2_ and 70 mM SrCl_2_ (cations known to form stronger alginate networks than calcium). These concentrations were selected based on previous experience with BaCl_2_ crosslinking and cell-free trials to determine stable capsule parameters for BaCl_2_ and SrCl_2_ crosslinking indicated by highest average circularity measurements (figures S13 and 14) [10]. Both supported aggregate formation and sustained growth throughout differentiation (figure 3(D)). Phase images of each day of differentiation are shown in figures S15 (strontium) and S16 (barium). Aggregate loss was markedly reduced in these conditions, with BaCl_2_ crosslinked capsules exhibiting minimal aggregate loss. Additionally, higher affinity crosslinkers produced smaller capsule diameters (453 ± 28 *μ*m for SrCl_2_ versus 359 ± 20 *μ*m for BaCl_2_), at identical printing parameters, consistent with greater network contraction during gelation.

In order to quantitatively assess aggregate retention, the average number of aggregates per capsule was monitored throughout differentiation (figure 3(E)). At the onset of differentiation, capsules crosslinked in 200 mM CaCl_2_ and 70 mM SrCl_2_ contained significantly more aggregates than the BaCl_2_ condition, which exhibited approximately half of that of the other conditions (figure 3(E)). By Day 5, both CaCl_2_ and SrCl_2_ conditions showed substantial aggregate loss, likely reflecting decapsulation, whereas BaCl_2_ capsules maintained a consistent aggregate count. By day 11 there were no significant differences in the number of aggregates when BaCl_2_ was compared to either 200 mM CaCl_2_ or 70 mM SrCl_2_. Significant values at the start and end of differentiation are noted to emphasize differences in initial and terminal states, though other comparisons of groups during differentiation may also be significant. Substantial variability in aggregate numbers was observed within all conditions, suggesting that printing at 5 l min^−1^ flow rate does not guarantee a consistent and reproducible aggregate generation rate.

Aggregate size dynamics were next examined for retained aggregates, by measuring aggregate diameters from each condition and on each day of differentiation, (figure 3(F)). Again, significant comparisons at the start and end of differentiation are noted to demonstrate differences between initial and terminal states, though other comparisons of groups during differentiation may also be significant. To determine the mechanistic basis of these cell dynamics, we further developed a logistic growth model (detailed in 3.5.1)

#### 3.3.2. Differentiated aggregates in all crosslinking conditions remain viable and express TEPC markers

Although decapsulation occurred to varying extents, retained aggregates across all conditions showed minimal debris accumulation during culture (figure S17). Upon harvesting on Day 11, aggregates retained their morphology and viability across conditions (figure 4(A)). Quantitative Live/Dead analysis confirmed robust survival, with average live area of 92.6% and 88.6% for 100 mM and 200 mM CaCl_2_ respectively (figure 4(B)). Dead cell distribution showed no significant differences between conditions, with dead cells primarily localized around the periphery of the aggregate, consistent with normal cell culture debris (figure 4(C)).

**Figure 4.**
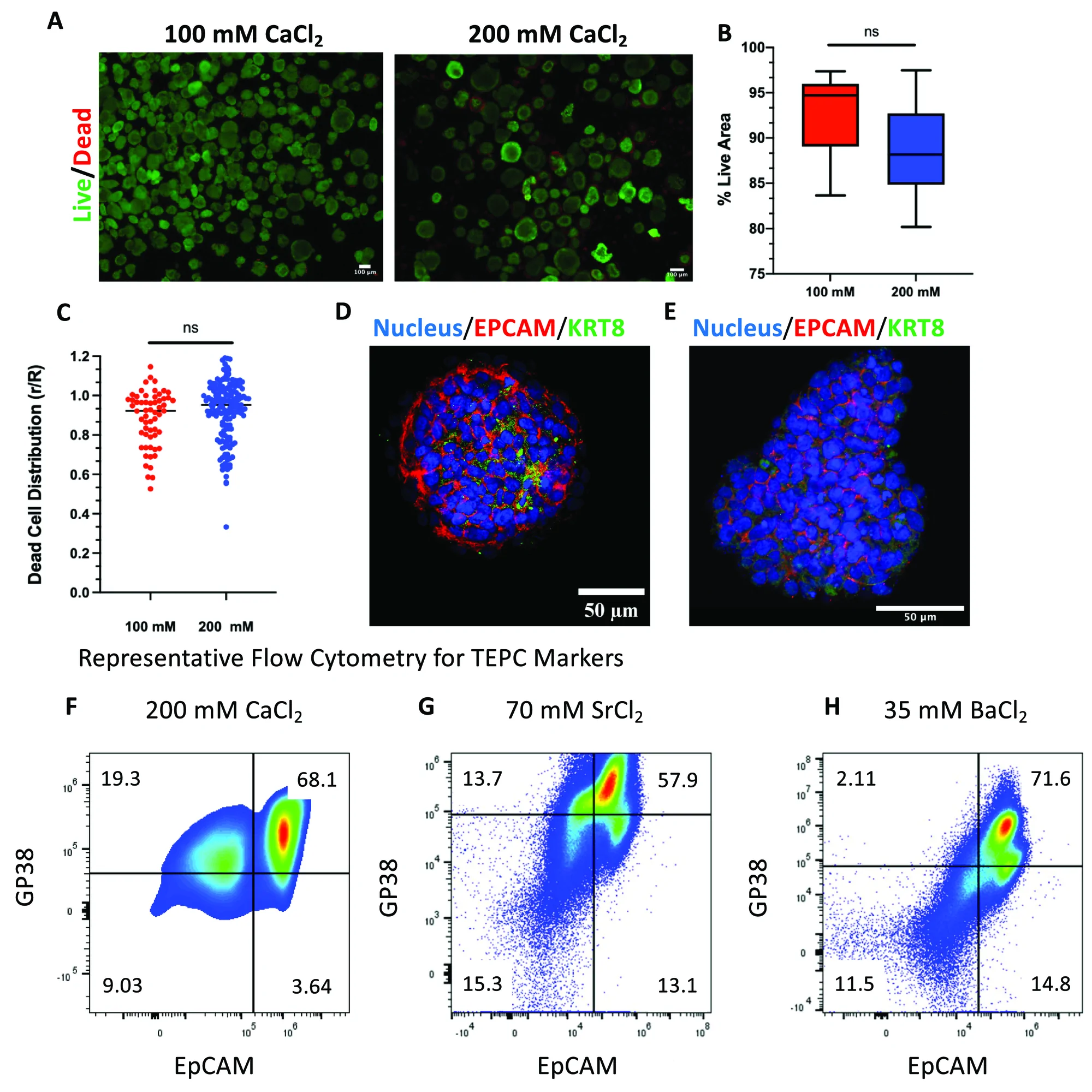
(A) Live/Dead staining of aggregates differentiated in alginate capsules crosslinked in 100 mM or 200 mM CaCl_2_ on Day 11 of thymic epithelial progenitor cell (TEPC) differentiation. Live cells are shown in green, dead cells are shown in red. Scale bar represents 100 *μ*m. (B) Area percentage of iPSC-TEPC aggregates (crosslinked in either 100 mM or 200 mM CaCl_2_) represented by viable cells, as indicated by green signal in Live/Dead staining (*n* = 10). ns indicates non-significance. (C) Location of dead cells in the aggregates shown in (A) as indicated by red signal in Live/Dead staining. *r*/*R* of 0.0 indicates the center of the aggregate, and an *r*/*R* of 1.0 or greater indicates the edge of the aggregate (n = minimum 59 cells). ns indicates non-significance. (D), (E) Printed TEPC aggregates after TEPC differentiation were fluorescently stained for nuclei (DAPI), TEPC marker KRT8 (green), and TEPC marker EpCAM (red). ((D): crosslinked in 100 mM CaCl_2_ at 5 l min^−1^ (E): crosslinked in 200 mM CaCl_2_ at 5 l min^−1^) Scale bar representing 50 *μ*m. (F–H) Printed TEPC aggregates crosslinked in (F) 200 mM CaCl_2_ (G) 70 mM SrCl_2_ (H) 35 mM BaCl_2_ (all at 5 l min^−1^) were characterized with flow cytometry for EpCAM and GP38, key epithelial TEPC markers delineating cortical TEPCs (EpCAM^low^GP38^high^) from medullary TEPCs (EpCAM^high^GP38^low^). Shown is a representative graph of expression.

Immunostaining confirmed successful TEPC differentiation across conditions. Aggregates from both the conditions positively expressed EpCAM, a key epithelial marker (figures 4(D) and (E)). Subsequent staining for a cTEC marker, KRT8, revealed that while both conditions expressed KRT8 protein, the 100 mM CaCl_2_ exhibited a stronger expression compared to the 200 mM (figures 4(D) and (E); quantified in figure S18).

The effect of crosslinking chemistry on TEC phenotype was then tested with Flow Cytometry analysis (FCM) for EpCAM. Immunostaining and FCM analysis of the bioprinted TEPC aggregates crosslinked with CaCl_2_, SrCl_2_ and BaCl_2_ revealed overall high population of EpCAM^high^ under all the tested conditions. Crosslinking with CaCl_2_ and SrCl_2_ exhibited 72% and 71% EpCAM^high^ expression respectively while BaCl_2_ showed 86% expression (figures 4(F)–(H)). When GP38 expression is considered alongside EpCAM expression populations specific to mTEC and cTEC lineages emerge in a crosslinker dependent manner [34]. CaCl_2_ shows a distinct population (19.3%) of EpCAM^low^GP38^high^ cells which is consistent with the cTEC lineage, while BaCl_2_ yields a distinct population (14.8%) of EpCAM^high^GP38^low^ cells consistent with the mTEC lineage (figures 4(F) and (H)). SrCl_2_ on the other hand displayed equal populations of cTEC and mTEC lineages with 13.7% and 13.1% expression respectively (figure 4(G)). Thus, the above analysis indicates successful differentiation across all the bioprinting conditions, with some effect on differentiation efficiency and lineage specification based on crosslinking chemistry.

### 3.4. Decreasing airflow rate increases capsule size and improves consistency of bioprinted TEPCs

#### 3.4.1. Increased capsule volume enhances aggregate retention

Although increasing the strength of the crosslinker cation decreased the extent of early aggregate decapsulation, aggregate loss was not fully eliminated, thereby affecting the final yield. We hypothesized that increasing the capsule volume, achieved by decreasing the airflow rate, would provide more space for aggregate growth, thereby enhancing retention and consistency. While the same seeding density of cells is used regardless of airflow rate, the total number of aggregates does not linearly increase with increased capsule size. This allows more volume for each aggregate to occupy. Reducing airflow rate from 5 to 2 l min^−1^ substantially increased mean capsule diameters across all crosslinking conditions; BaCl_2_ crosslinked capsules reached 1187 ± 73 *μ*m, SrCl_2_ capsules 1204 ± 72 *μ*m, and 200 mM CaCl_2_ capsules 1062 ± 26 *μ*m.

To accommodate the larger capsule geometry, the expansion phase was extended to 3 d, to allow for further aggregate growth prior to TEPC differentiation. This approach dramatically reduced decapsulation across all conditions. CaCl_2_ crosslinked capsules showed marked improvement over the 5 l min^−1^ condition, though some aggregate loss still persisted. The use of SrCl_2_ crosslinking further reduced aggregate loss, while BaCl_2_ crosslinking achieved near-complete aggregate retention indicated by the absence of free aggregates outside of capsules (figure 5(A)). Supporting phase contrast imaging spanning the differentiation protocol is provided in figures S19–S21. Live/Dead staining confirmed high aggregate viability across all conditions, at the end of TEPC differentiation. Aggregates maintained structural integrity following harvesting from the capsule with EDTA treatment, with only minimal peripheral cell debris observed (figures 5(B) and S22).

**Figure 5.**
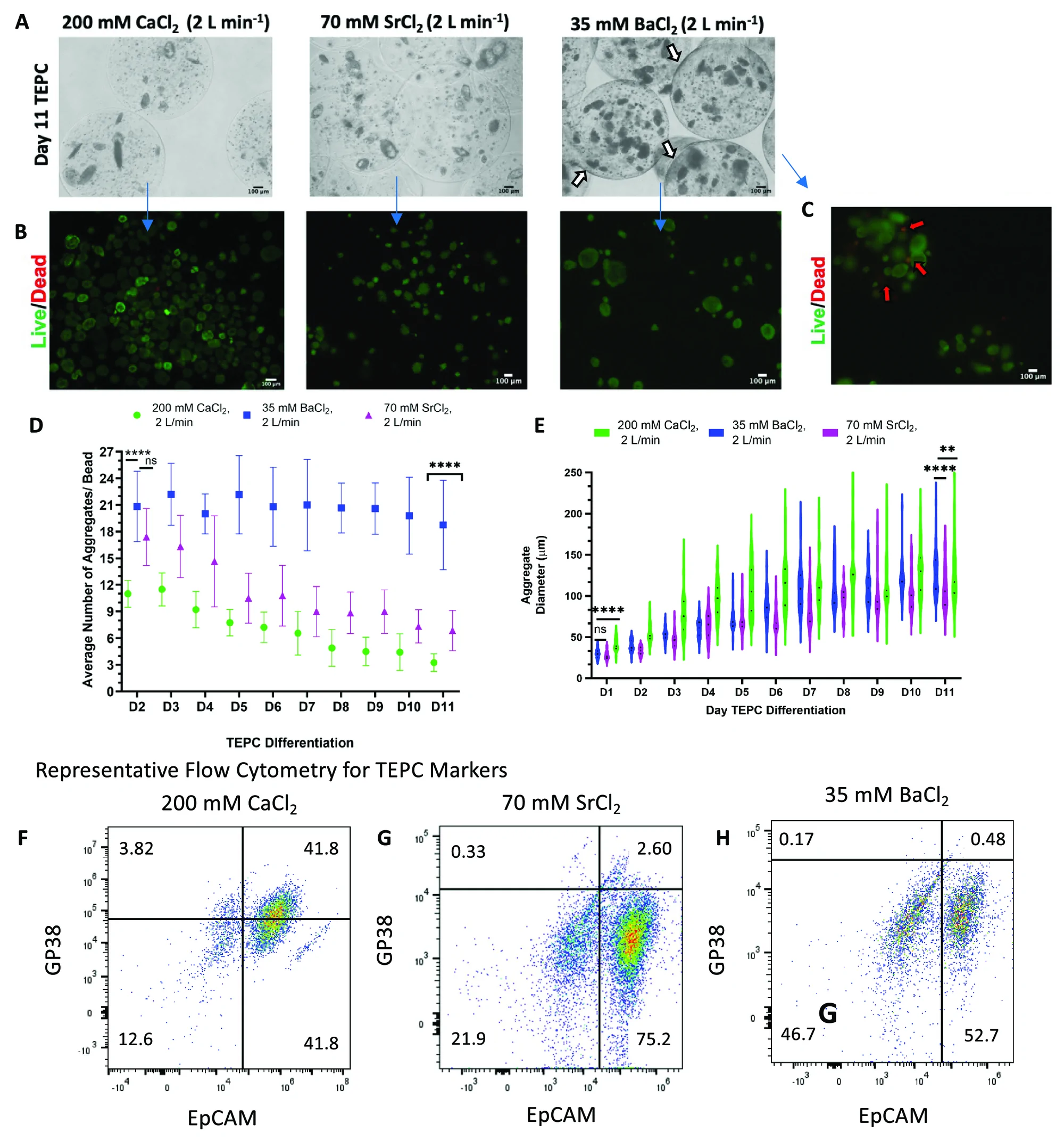
(A) Phase images of printed TEPC aggregates crosslinked in 200 mM CaCl_2_ (left), 70 mM SrCl_2_ (center), or 35 mM BaCl_2_ (right) and printed with an airflow speed of 2 l min^−1^. White arrows indicate chemical precipitates (B) Live/Dead staining of TEPC aggregates corresponding to conditions presented in (A). Imaging was conducted after the alginate capsules were dissolved and aggregates were decapsulated. Live cells are shown in green, dead cells are shown in red. Scale bar represents 100 *μ*m. (C) Live/Dead staining of encapsulated 35 mM BaCl_2_, imaged within the capsule. Scale bar represents 100 *μ*m. Red arrows indicate examples of dead cells. (D) Average number of aggregates/capsule and (E) Average aggregate diameter at specific days of TEPC differentiation for capsules crosslinked in 200 mM CaCl_2_, 70 mM SrCl_2_, and 35 mM BaCl_2_ at 2 l min^−1^ airflow speed (*n* = minimum 5 capsules). Significance values are noted at the beginning and end, though other comparisons may also be significant. (**: *p* < 0.01, ****: *p* < 0.001, ns: not significant). (F–H) Flow cytometry characterization of printed TEPC aggregates characterized with FCM for EpCAM and GP38, key epithelial TEPC markers delineating cortical TEPCs (EpCAM^low^ GP38^high^) from medullary TEPCs (EpCAM^high^GP38^low^). (F) crosslinked in 200 mM CaCl_2_ (2 l min^−1^) (G) crosslinked in 70 mM SrCl_2_ (2 l min^−1^) and (H) crosslinked in 35 mM BaCl_2_ (2 l min^−1^).

Worth noting, when BaCl_2_-crosslinked capsules contacted TEPC differentiation media, dark particulates formed throughout the hydrogel matrix, with pronounced accumulation at the capsule peripheries (figure 5(A): 35 mM BaCl_2_, white arrows demonstrate location of precipitates). Control experiments using cell-free BaCl_2_ crosslinked capsules confirmed these particles represented chemical precipitates rather than cellular debris (figure S23). Live/Dead staining demonstrated when dead cell clusters are present, they are located adjacent to live aggregates away from capsule periphery (figure 5(C), red arrows demonstrate location of dead cells).

Quantitative tracking of aggregate numbers revealed striking differences in retention efficiency (figure 5(D)). CaCl_2_ crosslinked capsules lost 70% of initial aggregates, whereas BaCl_2_ crosslinked capsules retained 90% of aggregates. This enhanced retention directly translated to efficient and predictable aggregate yield by the end of differentiation. Significant values at the start and end of differentiation are noted to demonstrate differences between initial and terminal states, though other comparisons of groups during differentiation may also be significant.

#### 3.4.2. Capsule geometry influences phenotypic specification

Temporal analysis of aggregate growth dynamics confirmed crosslinker-dependent effects on expansion kinetics (figure 5(E)). By Day 11 of differentiation SrCl_2_ encapsulated aggregates were consistently smaller compared to both BaCl_2_ and CaCl_2_ conditions, which showed similar growth profiles. Again, significant comparisons at the start and end of differentiation are noted to demonstrate differences between initial and terminal states, though other comparisons of groups during differentiation may also be significant. Notably, all crosslinker conditions at 2 l min^−1^ produced significantly larger aggregates than their 5 l min^−1^ counterparts (figures S24(A)–(C) for Day 11) validating our hypothesis that increased capsule volume enables larger individual aggregate growth.

Finally, we assessed differentiation efficiency by TEPC marker expression via flow cytometry. With CaCl_2_ condition, we achieved 84% EpCAM^high^ (figure 5(F)). Similarly, SrCl_2_ also achieved high EpCAM^high^ expression at 78% (figure 5(G)). Both are similar to what was seen for 5 l min^−1^ printing for these crosslinkers. Conversely, BaCl_2_ crosslinking yielded only 53% EpCAM^high^ cells, which was decreased from the 5 l min^−1^ airflow (figure 5(H)). A comparison of the EpCAM^high^, and cTEC and mTEC percentages between the 2 l min^−1^ and 5 l min^−1^ printing airflow rates for the crosslinkers assessed with flow cytometry is included in Supplemental figures S25(A)–(C).

When GP38 expression was considered for lineage specification, mTEC populations (EpCAM^high^GP38^low^) were the dominant phenotype expressed, with much lower populations of cTECs compared to printing at 5 l min^−1^ (figures 5(F)–(H), S25(B) and (C)). Interestingly, in the 2 l min^−1^ printing with SrCl_2_ both populations no longer appear in similar numbers as in the 5 l min^−1^ case with the specification largely to mTEC (75.2%) (figure 5(G)).

#### 3.4.3. Optimizing the tradeoff between throughput and reproducibility

We evaluated production efficiency across all tested conditions (table 2). At the higher airflow rate 5 l min^−1^, the aggregate generation rates were high, ranging from 26.0 ± 23.5 × 10^3^ aggregates/ml bioink (100 mM CaCl_2_) to 83.7 ± 49.9 × 10^3^ aggregates/ml (35 mM BaCl_2_). However, these conditions exhibited substantial batch-to-batch variability, with CV ranging from 0.528 to 0.902, indicating poor reproducibility.

**Table 2.** Production and density schematic of TEPC aggregates crosslinked in varying conditions and printed at varying airflow speeds. The alginate concentration for all conditions was 2.0%.

Crosslinker	Airflow speed (l min^−1^)	D11 # Aggregates/bead	Aggregates generated/ml bioink (10^3^)	Coefficient of variance (CV)
100 mM CaCl_2_	5	1.33 ± 1.20	26.0 ± 23.5	0.902
200 mM CaCl_2_	5	2.67 ± 1.41	66.4 ± 35.0	0.528
70 mM SrCl_2_	5	1.57 ± 0.96	32.3 ± 19.7	0.611
35 mM BaCl_2_	5	2.03 ± 1.21	83.7 ± 49.9	0.596

200 mM CaCl_2_	2	3.25 ± 0.99	5.2 ± 1.6	0.305
70 mM SrCl_2_	2	6.87 ± 2.26	7.5 ± 2.5	0.238
35 mM BaCl_2_	2	18.75 ± 5.02	21.5 ± 5.8	0.268

Reducing the airflow to 2 l min^−1^ decreased absolute production rates by approximately one order of magnitude but dramatically improved consistency, with all conditions achieving CV values below 0.33. The relationship between crosslinker binding affinity and aggregate retention became particularly pronounced at this lower airflow rate. While 200 mM CaCl_2_ produced only 3.25 ± 0.99 aggregates/capsule at Day 11, SrCl_2_ produced 6.87 ± 2.26 and BaCl_2_ produced 18.75 ± 5.02—a nearly 6-fold improvement achieved by increasing cation-alginate binding strength.

### 3.5. Logistic growth modeling reveals biophysical constraints governing aggregate expansion and informs parameter optimization

Given the number of tunable parameters, we wished to investigate the entire printing parameter space, and identify optimal conditions for reproducible production. We applied logistic growth modeling to the aggregate growth dynamic data collected over time for all material and airflow conditions (figures 3(F) and 5(E)). Fitting to the mean aggregate size for each day of differentiation resulted in the best model (average *R*^2^ of 0.98 compared to fit to individuals with average *R*^2^ of 0.67) and further analysis was completed using this fit using both raw data values (figures 6(A)–(C)), and growth values normalized to max aggregate size (figures 6(D)–(F)).

**Figure 6.**
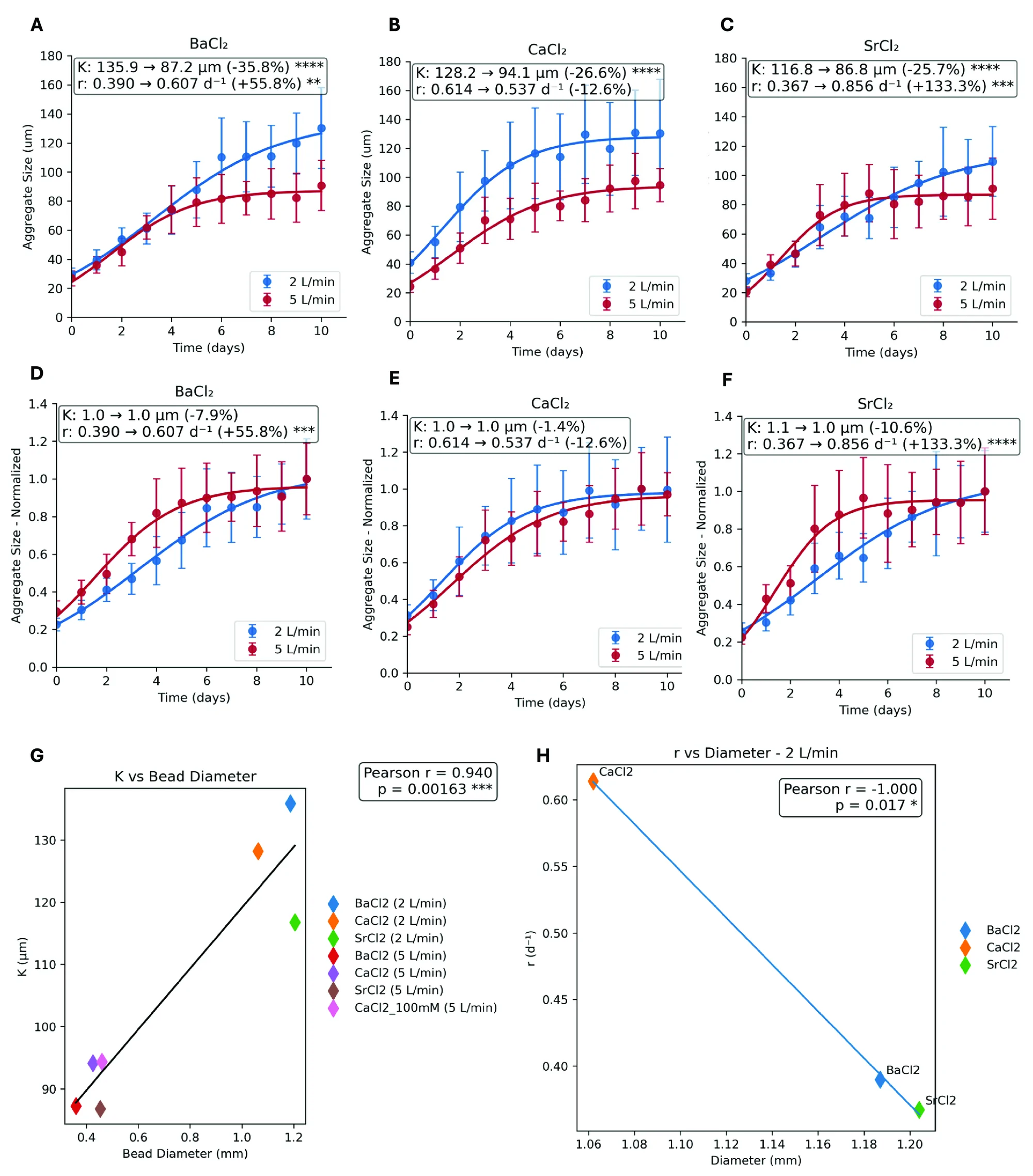
(A)–(C) Fits for each crosslinker and each printer airflow rate and (D)–(F) aggregate size measurements normalized to max mean aggregate size calculated across days of differentiation. Figure annotation shows change in model parameters from 2 to 5 l min^−1^ condition with percent change in parameters noted. Wald testing was completed to detect significant differences in parameter changes (**: *p* < 0.01, ***: *p* < 0.005, **** *p* < 0.001). (G) Correlation and regression analysis performed for all values of *K* vs average capsule diameter. Pearson *r* was calculated for the correlation analysis with a *t*-test performed to assess significance (***: *p* < 0.005). (H) Correlation and regression analysis performed for values of *r* at 2 l min^−1^ airflow rate vs average capsule diameter. Pearson *r* was calculated for the correlation analysis with a *t*-test performed to assess significance (*: *p* < 0.05).

#### 3.5.1. Capsule geometry constrains carrying capacity

Carrying capacity was significantly higher at 2 l min^−1^ compared to 5 l min^−1^ across all crosslinkers (BaCl_2_ 135.9 vs 87.2 *p* < 0.001, CaCl_2_ 128.2 vs 94.1 *p* < 0.001, SrCl_2_ 116.8 vs 86.8 *p* < 0.001) (figures 6(A)–(C)). When fitted values for *K* were paired with average capsule diameter for these conditions, it was found that increasing capsule diameter was directly correlated with carrying capacity (Pearson *r* = 0.94, *p* = 0.001) (figure 6(G)), indicating that capsule volume directly limits maximum aggregate size. This geometric constraint likely reflects the physical space available for expansion within the alginate matrix.

#### 3.5.2. Spatial confinement accelerates growth kinetics

Paradoxically, while smaller capsules limited final aggregate size, they promoted faster initial growth. In terms of growth rate, for the BaCl_2_ and SrCl_2_ conditions growth rate was significantly higher in the 5 l min^−1^ condition relative to the 2 l min^−1^ condition (BaCl_2_ 0.61 vs 0.39 *p* = 0.006, SrCl_2_ 0.86 vs 0.37 *p* < 0.001) (figures 6(a) and (C)). This effect is particularly evident when normalizing the aggregate trajectories to the maximum aggregate size, which shows a clear increase in the initial rate of growth in smaller capsules, despite their reduced carrying capacity (figures 6(D)–(F)). CaCl_2_ conditions showed no significant airflow-dependent change in growth rate (0.54 vs 0.61 *p* = 0.447) (figure 6(B)). This suggests crosslinker-specific modulation, which will need to be investigated further in future studies.

Interestingly, when the percentages of cTEC and mTEC like populations were plotted for both 2 l min^−1^ and 5 l min^−1^ airflow printing rates, 5 l min^−1^ airflow rates promoted higher percentages of cTECs (figure S25(A)) while 2 l min^−1^ promoted higher percentages of mTECs (figure S25(B)). As mentioned above, the smaller capsule size is associated with higher initial growth rates. This indicates capsule size may not only affect the growth rate of the TEPC aggregates but also their phenotypic specification.

Notably, when capsule diameter is plotted against the *r* values for 2 l min^−1^ conditions, there is a strong negative correlation between capsule size and growth rate (Pearson *r* = −1.0 *p* = 0.017) (figure 6(H)). While this correlation is diminished when considering all the capsule diameters and aggregate growth rates in the 2 l min^−1^ and 5 l min^−1^ conditions, the trend is still present (Pearson *r* = −0.67 *p* = 0.103) (figure S26). We speculate that this inverse relationship between capsule size and growth rate may indicate enhanced diffusion transport in smaller volumes, facilitating efficient nutrient delivery.

To demonstrate this, we modeled diffusion in a spherical hydrogel capsule of radius *R* and solved for the analytical concentration profile from diffusion at the spherical boundary using Fick’s second law. Ultimately this demonstrates that the maximum sustainable cellular demand is inversely proportional to the square of the capsule radius. In other words, smaller capsules promote higher cellular consumption which may result in increased growth rate. Additional details of this analysis are included as part of the supplemental information (Steady-State Reaction-Diffusion in a Spherical Capsule.pdf). These analytical findings corroborate the result we obtained through logistic modeling.

### 3.6. Printed TECs can support T cell selection *in vivo*

We further conducted an *in vivo* study to confirm functionality of our manufactured TEPCs. Functionality was assessed through the ability of implanted TEPCs to facilitate T cell selection in nude mice. Briefly, T cell selection is a stepwise process that is dependent on the different lineages of TECs in the thymus. Circulating Pro-Ts enter the thymus while they are double negative for CD4 and CD8 expression (figure 7(A)). First, they interact with cTECs in the thymic cortex where they follow a series of developmental checkpoints including T cell lineage specification and TCR beta chain selection to become CD4^+^CD8^+^ double positive cells in a process termed positive selection. Following positive selection, double positive cells enter the thymic medulla where they undergo negative selection and commit to either the CD4 (helper) or CD8 (cytotoxic) T cell lineage. At this single positive stage, mature T cells exit the thymus and are transported to peripheral organs such as the spleen and lymph nodes.

**Figure 7.**
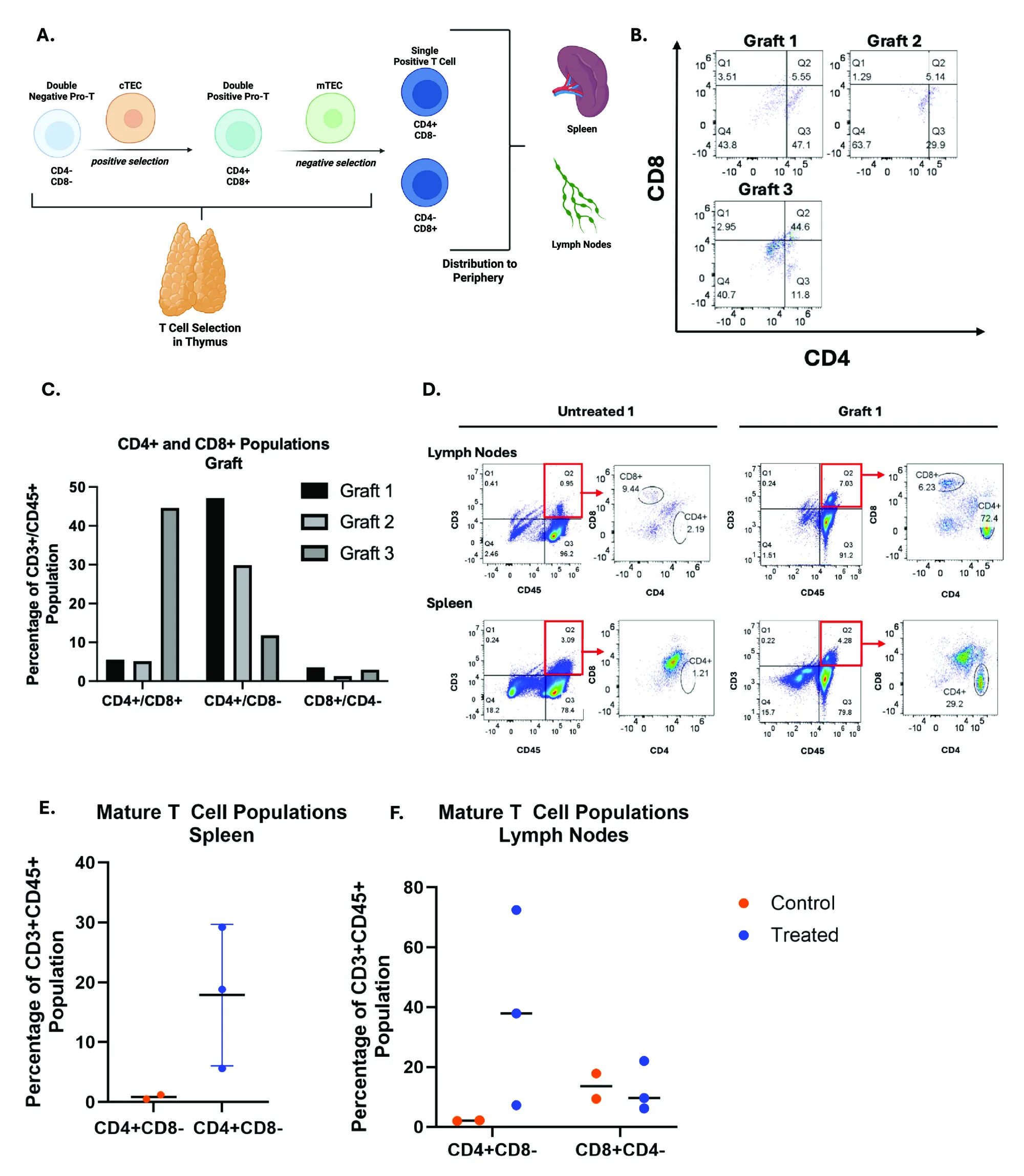
(A) Schematic diagram describing the process of T cell selection. Pro-Ts enter the thymus as double negative for CD4 and CD8. Following positive selection by cortical thymic epithelial cells (cTECs) Pro-Ts become double positive for CD4 and CD8. Following negative selection by medullary thymic epithelial cells (mTECs), double positive T cell progenitors commit to either a CD4^+^ or CD8^+^ lineage and exit the thymus for peripheral organs. Created in BioRender. Parlow, E. (2026) https://BioRender.com/surmzy0 (B) Flow cytometry analysis demonstrates that successful programming of murine Pro-Ts was achieved in the graft, which resulted in emergence of CD4^+^CD8^+^, CD4^+^CD8^−^, and CD8^+^CD4^−^ populations (C) Percentage of murine CD3^+^CD45^+^ cell population expressing CD4 or CD8 markers in each of the harvested grafts. (D) Comparison of murine CD4^+^, and CD8^+^ populations in untreated and graft implanted lymph nodes and spleens. One representative individual in each population is shown. In the lymph nodes, both murine CD4^+^ and CD8^+^ populations are distinct while in the spleen only the CD4^+^ appears distinct. (E) Percentage of murine CD3^+^CD45^+^ cell population that are CD4^+^CD8^−^ in the harvested spleens for both control and treated mice. (F) Percentage of murine CD3^+^CD45^+^ that are CD4^+^CD8^−^ or CD8^+^CD4^−^ in the harvested lymph nodes for both control and treated mice.

TEPCs used for the study were printed using 2.0% alginate, 2 l min^−1^ airflow speed, crosslinked in 200 mM CaCl_2_, and differentiated using the standard TEPC differentiation protocol. CaCl_2_ was selected as the crosslinker due to previous experience with *in vivo* studies [15]. Given the lower coefficient of variability and larger bead size, we elected to pursue the 2 l min^−1^ airflow rate. iPSC-TEPC scaffolds were constructed by seeding decapsulated TEPC aggregates into a decellularized mouse scaffold along with *in vitro* generated human Pro-Ts to promote TEPC maturation. Scaffolds were cultured *in vitro* for 7 d and were then implanted beneath the kidney capsule. The control mice did not receive an implant. After 45 d, mice were sacrificed, and the graft, spleen, and mesenteric lymph nodes were harvested and digested. Single cells were stained for murine lymphoid and T cell markers.

Evidence of functional T cell selection is demonstrated through populations of murine CD4^+^CD8^+^ double positive thymocytes located in the graft (figures 7(B) and (C)). Graft 3 showed a high (44.6%) percentage of CD4^+^CD8^+^ cells in the graft, possibly suggesting a robust functionality compared to other treated mice. All the grafts showed populations of CD4^+^CD8^−^ and CD8^+^CD4^−^ T cells. The outcome of the T cell selection in the graft was towards high percentages of CD4^+^ cells relative to CD8^+^ cells (29.6% vs 2.58%). Overall, the presence of double positive and mature T cells in the grafts suggests both functionality of the selecting iPSC-TEPCs and the capacity of circulating murine Pro-Ts to home to the thymus graft to undergo selection.

In addition, once selection was completed data from peripheral organs show a systemic effect of the implanted thymus grafts. Digested tissue samples underwent flow cytometry and were first gated to isolate murine CD45^+^ CD3^+^ positive populations. CD45 represents the common leukocyte antigen and is used to isolate populations of leukocytes, while CD3 is specific to T cells. From this population, sub-populations of murine CD4 and CD8 positive cells were identified. When compared to untreated mice, mice that received the graft showed emergence of a population of CD4^+^CD8^−^ cells in both the lymph nodes and the spleen (figures 7(D)–(F) and S27). In both the spleen and the lymph nodes, the percentage of CD4^+^CD8^−^ cells was more pronounced relative to the untreated control (figures 7(D)–(F)). In the spleen, the average expression of CD4 single positives was 17.9% for treated mice and 0.86% in the control mice, while in the lymph nodes the average expression of CD4 single positives was 39.8% versus 2.10% for controls. Emergence of a distinct CD8^+^CD4^−^ population was not seen in the spleen for either the graft or the untreated mice, but in the lymph nodes a similar percentage of these cells existed for both groups (12.6% graft versus 13.6% control) (figure 7(F)).

## 4. Discussion

### 4.1. Scalability and droplet bioprinting

The transition from laboratory-scale organoid generation to clinical manufacturing is currently hindered by the lack of scalable bioprocessing technologies capable of meeting the estimated demand of billions of cells per tissue construct [16]. While suspension bioreactors, such as stirred-tank systems, are the standard for expanding robust cell types, iPSCs present unique challenges due to their sensitivity to hydrodynamic shear stress [17–20]. High shear rates in these vessels often lead to heterogeneous cultures and reduced differentiation efficiency, and while low-shear alternatives like Vertical Wheel bioreactors exist, they remain cost-prohibitive for widespread single-use implementation [35–37]. Similarly, static 3D culture methods, including microwell arrays and hanging drop plates, are inherently limited by surface area and labor-intensive handling, capping production at approximately 5800 spheroids per plate even with robotic automation [38–40]. The Kugelmeiers Sphericalplate 5D^TM^ has emerged as an option to increase the total number of aggregates, achieving up to 9000 spheroids per plate, but ultimately still functions in the same way as a microwell and is limited by surface area and handling [41, 42]. While these systems may be able to establish greater homogeneity in the size of the cultured spheroids, in the current study size heterogeneity did not seem to compromise the ultimate *in vivo* functionality.

Bioprinting has emerged as a strategy to automate this process, however, standard extrusion methods often fail to achieve the necessary throughput. For instance, Lawlor *et al* demonstrated the utility of printing iPSCs as a high-density cell paste for kidney organoids, yet this approach was limited to a production rate of roughly 18 organoids per minute [21]. Our study demonstrates that air-jet droplet bioprinting overcomes these limitations by decoupling droplet generation from mechanical extrusion force. By utilizing a coaxial air stream to atomize a low-viscosity, single-cell alginate suspension, we achieved a production rate of approximately 5.0 droplets per second (1500 capsules per 5 min) using the optimized 2 l min^−1^ airflow parameter. This represents a magnitude of production comparable to or exceeding other leading droplet generation studies, such as Shen *et al*, who generated ∼3.3 capsules per second via electrostatic printing [43], and Lee *et al*, who reported ∼4 capsules per second using a similar air-jet mechanism [44].

Critically, this increase in throughput does not come at the cost of biological function. The droplet printing approach creates microscale capsules with a high surface-to-volume ratio, facilitating the efficient transport of oxygen and nutrients essential for maintaining high cell density aggregates. By optimizing the system to print at 2 l min^−1^, we were able to generate a consistent yield of 21 500 aggregates per ml of bioink. This establishes air-jet droplet bioprinting as a robust, cost-effective platform that bridges the gap between the precision of micro-scale engineering and the volume requirements of clinical therapy, offering a viable alternative to both shear-intensive bioreactors and low-throughput extrusion systems.

### 4.2. Hydrogel crosslinking chemistry governs aggregate retention while maintaining differentiation competence

The physical stability of the bioprinted microenvironment proved to be a decisive factor in the successful generation of TEPC organoids, a property largely governed by the rheology of the bioink and the crosslinking chemistry of the hydrogel network. While droplet generation physics are controlled by airflow, the biological parameters, specifically aggregate retention and differentiation, are dictated by the resilience of the biomaterials in a physiological environment.

We observed that the inclusion of cells significantly alters the rheological properties of the alginate: low concentration bioinks (1.6% w/v) failed to form stable capsules upon cellular inclusion, likely due to the modification of bioink viscosity from the inclusion of cell slurry. Increasing the alginate concentration to 2.0% w/v restored the shear-thinning behavior necessary for stable droplet formation, while the long-term integrity of these capsules was heavily dependent on the crosslinking cation.

A significant challenge identified in this study was ‘decapsulation,’ where growing aggregates breached the hydrogel boundary. Decapsulation was measured through visual inspection and assessment of the average number of aggregates in alginate capsules over time. This phenomenon was more prevalent in calcium crosslinked capsules. Alginate gelation is known to be sensitive to even slight changes in pH. Decapsulation was most prominent in the window after switching of base media from Stage I to Stage II base media, which may coincide with a pH change. Notably, when BaCl_2_ was substituted as the crosslinker, decapsulation was greatly reduced. This observation is consistent with previous studies that demonstrated barium crosslinked particles tend to be more resistant to pH associated deformation than those crosslinked with calcium [45].

Modification of alginate concentration, crosslinking chemistry, and concentration results in modulation of substrate mechanical properties, which can further influence the phenotype of the encapsulated cells, as we have shown previously [10]. For example, increasing barium chloride crosslinking concentration from 10 mM to 100 mM resulted in a significant (∼18-fold) increase in the Young’s modulus, from 3.9 ± 1.3 kPa to 73.3 ± 22.4 kPa, as measured by AFM nanoindentation methods [10]. The choice of crosslinking cation is also important. Mørch *et al* report that at a given concentration SrCl_2_ hydrogels exhibit stiffnesses approximately 1.67 times higher than CaCl_2_ hydrogels, while BaCl_2_ hydrogels are approximately 15 times stiffer [46]. Although such modifications of the biophysical microenvironment are likely to affect differentiation efficiency of the TEPCs, our bioink parameters were selected to preserve differentiation and functionality while focusing on maximizing throughput and reproducibility.

### 4.3. Manufacturability of iPSC organoids is impacted by biophysical and chemical constraints with a tradeoff between throughput and reproducibility

When the data for aggregate size was used to fit a logistic model, additional biophysical constraints that governed aggregate expansion emerged. Increasing capsule diameter correlated with higher carrying capacity, suggesting that larger capsules can tolerate larger terminal aggregate size. However, smaller capsules resulted in faster initial growth which may indicate enhanced diffusive transport in smaller capsule volumes. This inverse relationship reveals a fundamental confinement-driven trade off, as was also explained with the Fickian Diffusion model, with spatial constraint accelerating early kinetics while limiting terminal aggregate size, with direct implications for balancing throughput against organoid robustness.

In our previous work we have successfully integrated mathematical modeling with experimental stem cell biology to extract mechanistic insights from complex differentiation processes including under mechanical induction of biophysical cues [31, 33, 47, 48]. The logistic framework employed here extends this philosophy further to encapsulated organoid cultures. Our earlier work demonstrated that stem cell fate is sensitive to substrate microstructure, with specific microstructural features correlating with lineage-specific outcomes [33]. We further reported that culture configuration dominates proliferation and differentiation responses while substrate properties fine-tune expression within configuration-defined bounds [14]. The present findings are thus consistent with this hierarchy: capsule geometry, set by airflow-mediated droplet size, establishes the growth ceiling, whereas the crosslinker identity modulates the ensuing matrix properties to tune kinetic parameters within the specific geometric constraint. Although the current studies are not specifically focused on understanding the specific nuances of the mechano-chemical and mechano-biological aspects, future work will focus on decoupling the contributions of matrix stiffness, nutrient diffusion, and emergent cell–cell signaling. This will enable linking 3D printable process parameters directly to organoid quality attributes.

### 4.4. T cell programming and in vivo functionality

The ultimate validation of any bioengineered thymic construct lies in its capacity to facilitate the complex ‘handshake’ mechanisms required for T cell selection and lineage commitment. In the native thymus, this process is spatially orchestrated: Pro-Ts enter the cortical region where cTECs drive positive selection—ensuring T cells can recognize self-MHC—before migrating to the medulla for negative selection [1]. Our *in vitro* characterization confirmed that the air-jet bioprinting process generated aggregates with robust expression of EpCAM, and the cortical marker KRT8. Important to note here is that iPSCs generated from a male dermal fibroblast were used in all studies which are mesodermal in origin, while the target cell population, TEPCs are endodermal. Previous work has demonstrated that there are advantages to using lineage matched precursors to generate iPSCs for differentiation [49]. In the current study we limited ourselves to the same iPSC line that has provided efficient differentiation to thymic lineage in our previous report [15]. Our previous extensive characterization of the iPSC-TEC population from this cell line provides the basis to evaluate the performance of the current iPSC-TECs. The high percentage of EpCAM^high^ cells and subsequent success in *in vivo* studies demonstrate successful differentiation efficiency. While it will be interesting to explore the feasibility of enhancing differentiation efficiency using iPSCs generated from lineage matched organs, it is important to consider that doing so will restrict the translational potential of our technology because of lack of access to patient-specific endodermal organs.

We have previously reported the significant effect of alginate substrate stiffness on differentiation of encapsulated hPSCs towards the endoderm lineage [10]. Thymic epithelium originates from the third pharyngeal pouch endoderm, and iPSC-derived TEPC differentiation proceeds through definitive endoderm and foregut endoderm intermediates [3, 4], following the same developmental window through which pancreatic progenitors are specified. Thus, it is expected that the substrate mechanical properties will similarly affect the initial endoderm differentiation stage of the developing iPSC-TEPC population. Furthermore, recently Jeffreys *et al* engineered a thymic niche with an alginate-based interpenetrating network and showed that the matrix mechanics alone are sufficient to redirect the cell fate. Soft, stress-relaxing matrices sustained the T-cell development gene regulatory network and thereby accelerated progenitor T-cell differentiation. Meanwhile, stiffer, more elastic matrices suppressed T-lineage commitment and diverted hematopoietic progenitors toward the myeloid fate, acting through mechanosensitive Notch1 and integrin signaling [50]. While this work focused on the hematopoietic rather than the epithelial compartment it nevertheless indicates that the thymic microenvironment is mechanosensitive and that the alginate network mechanics are instrumental for mimicking such effects.

The critical role of a three-dimensional ECM microenvironment for TEC survival and function has been a central theme in our prior work. We demonstrated that decellularized mouse thymus scaffolds retain native ECM architecture and support TEC molecular identity for extended culture period, and when repopulated and transplanted into athymic nude mice, these organoids promoted thymopoiesis and enabled donor-specific immune tolerance [15, 25]. Our broader decellularization studies, spanning perfusion-decellularized pancreas [51, 52], organ-specific ECM arrays [9], and repopulation of decellularized scaffolds with human pluripotent stem cell-derived progenitor aggregates [53], have consistently shown that native ECM provides instructive cues that direct tissue-specific maturation, and that aggregate-based seeding achieves superior engraftment over single-cell approaches.

The present work integrates these two lines of investigation: scalable bioprinting within alginate capsules to generate TEPC aggregates, followed by decapsulation and transfer into decellularized mouse thymus scaffolds for *in vivo* implantation. While alginate lacks the instructive ligands of native thymic ECM, the encapsulation geometry promotes cell–cell aggregation and cortical niche establishment, as evidenced by KRT8 expression. The decellularized thymus scaffold then provides the three-dimensional ECM architecture necessary to support sustained TEC function upon transplantation. The recovery of a significant murine CD4^+^ single-positive T cell population in the engrafted mice indicates that the bioprinted TEPCs successfully recruited murine Pro-Ts and supported their transition through the critical CD4^+^CD8^+^ double-positive stage. While the murine CD8^+^ population remained relatively low compared to the CD4^+^ compartment, this CD4 bias is frequently observed in engineered thymic grafts and suggests that the bio-printed organoids predominantly established a cortical niche favoring MHC Class II restriction. Importantly, the generation of mature, single-positive CD4^+^ T cells confirms that the bioprinting process did not compromise the delicate antigen-presentation machinery of the epithelial cells. This demonstrates that scalable droplet bioprinting can produce organoids that not only physically resemble thymic tissue but are biologically competent to ‘train’ the adaptive immune system.

Among the various crosslinking conditions examined, we only evaluated the CaCl_2_-crosslinked construct *in vivo*. Notably, the BaCl_2_- and SrCl_2_-crosslinked constructs exhibited distinct differentiation biases during iPSC-TEPC differentiation *in vitro*. Future *in vivo* studies comparing these formulations will be crucial in determining whether the crosslinker-dependent effects on lineage specification persist *in vivo*. This information will be instrumental in optimizing the bioprinting conditions for further advancements.

## 5. Conclusion

Altogether we present a system that generates thymic epithelial organoids with high manufacturing throughput. We note how changes to process parameters impact output organoid quality. Most notably, we found that the reproducibility of printing is increased at lower airflow rates. Capsules printed at 2 l min^−1^ airflow rates had lower CVs in all crosslinking conditions. This comes at the expense of throughput, as 5 l min^−1^ conditions consistently produced more aggregates per ml of bioink, despite higher amounts of decapsulation. Chemical crosslinkers were key for preventing decapsulation of the aggregates. All crosslinkers were able to promote differentiation to the TEPC phenotype evidenced through high expression of EpCAM. Further, we confirmed functionality of printed TEPCs in *in vivo* models. Given the multiplicity of tunable parameters, our system will be useful for increasing manufacturability of iPSCs for a wide variety of applications.

## Data Availability

The data cannot be made publicly available upon publication because they contain commercially sensitive information. The data that support the findings of this study are available upon reasonable request from the authors. Steady-State-Reaction-Diffusion in a Spherical Capsule available at https://doi.org/10.1088/1758-5090/aeae75/data1. Supplemental Video 1 available at https://doi.org/10.1088/1758-5090/aeae75/data2. Supplemental Information available at https://doi.org/10.1088/1758-5090/aeae75/data3.
